# A case of jejunal cancer arising from ectopic pancreas

**DOI:** 10.1007/s12328-025-02148-5

**Published:** 2025-06-16

**Authors:** Makoto Eizuka, Yosuke Toya, Ryo Sugimoto, Mizuki Komai, Ako Yamada, Taku Kimura, Shunichi Yanai, Naoki Yanagawa, Akira Sasaki, Takayuki Matsumoto

**Affiliations:** 1 Division of Gastroenterology and Hepatology, Department of Internal Medicine, School of Medicine, Iwate Medical University, 2-1-1, Yahaba, Iwate 028-3695 Japan; 2 Department of Molecular Diagnostic Pathology, School of Medicine, Iwate Medical University, Yahaba, Iwate Japan; 3 Department of Surgery, School of Medicine, Iwate Medical University, Yahaba, Iwate Japan

**Keywords:** Jejunum, Cancer, Ectopic pancreas, Double-balloon endoscopy

## Abstract

A 74-year-old man presented with intestinal obstruction. CT (computed tomography) revealed a 25-mm mass in the proximal jejunum. Double-balloon endoscopy (DBE) identified a smooth, asymmetrically localized mass with oozing and luminal stenosis covered with the mucosa of swollen villi. Small bowel radiography demonstrated jejunal obstruction. Histopathological examination of the resected specimen confirmed moderately differentiated adenocarcinoma arising from ectopic pancreatic tissue of Heinrich type I. Our case suggests that cancer arising from ectopic pancreas should be considered as a candidate diagnosis of a submucosal tumor in the jejunum.

## Introduction

Ectopic pancreas is a developmental anomaly, which is characterized by the existence of pancreatic tissue in the gastrointestinal tract. It occurs a subepithelial lesion (SEL), particularly in the stomach and in the small intestine [1, 2]. As well as the normal pancreas, ectopic pancreas manifests pancreatitis, cystic formation, and bleeding, and, rarely, it transforms to cancer [3, 4].

We herein report a case of cancer arising from ectopic pancreas in the jejunum, which could be observed endoscopically.

## Case presentation

A 74-year-old man visited his neighboring hospital with anorexia, vomiting, and abdominal pain for a month. The patient did not have any specific medical history or medication. Because abdominal CT revealed small bowel dilation, he was admitted with a diagnosis of intestinal obstruction. Although his symptoms transiently relieved, he again complained of vomiting after he started the oral intake. Consequently, he was transferred to our hospital for further evaluation.

**Fig. 1:**
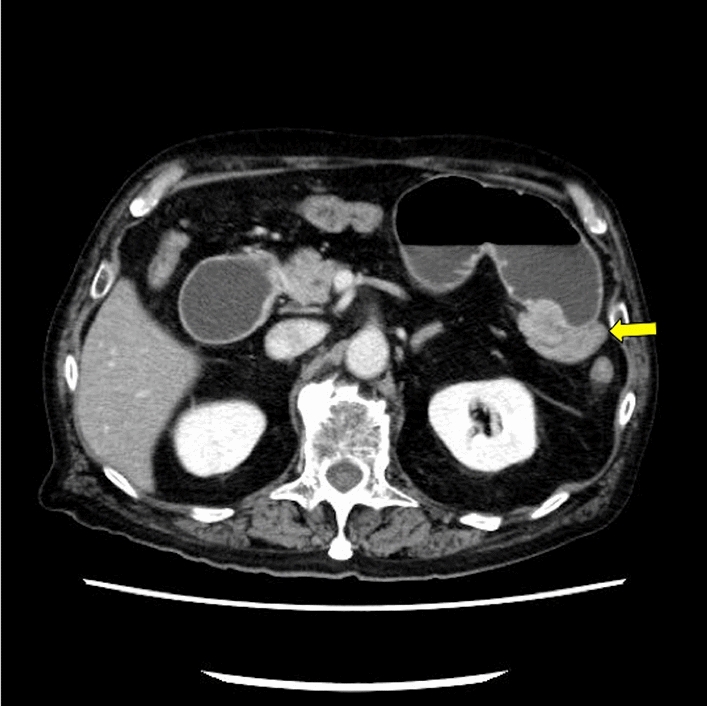
Contrast enhanced computed tomography revealed a 25 mm high-density mass in the jejunum (yellow arrow) with proximal bowel dilation

On admission, physical examination revealed abdominal distention and epigastric tenderness. Laboratory showed an elevated C-reactive protein (CRP) level of 0.88 mg/dl, an increased CA19-9 level of 684 ng/ml, and a low albumin level of 2.9 g/dl. Hemoglobin value was normal at level of 14.2 g/dl. Contrast enhanced CT identified a high-density mass measuring 25 mm in the jejunum distal to the ligament of Treitz, and proximal bowel dilation. Regional lymph nodes were not enlarged (Fig. 1).

**Fig. 2:**
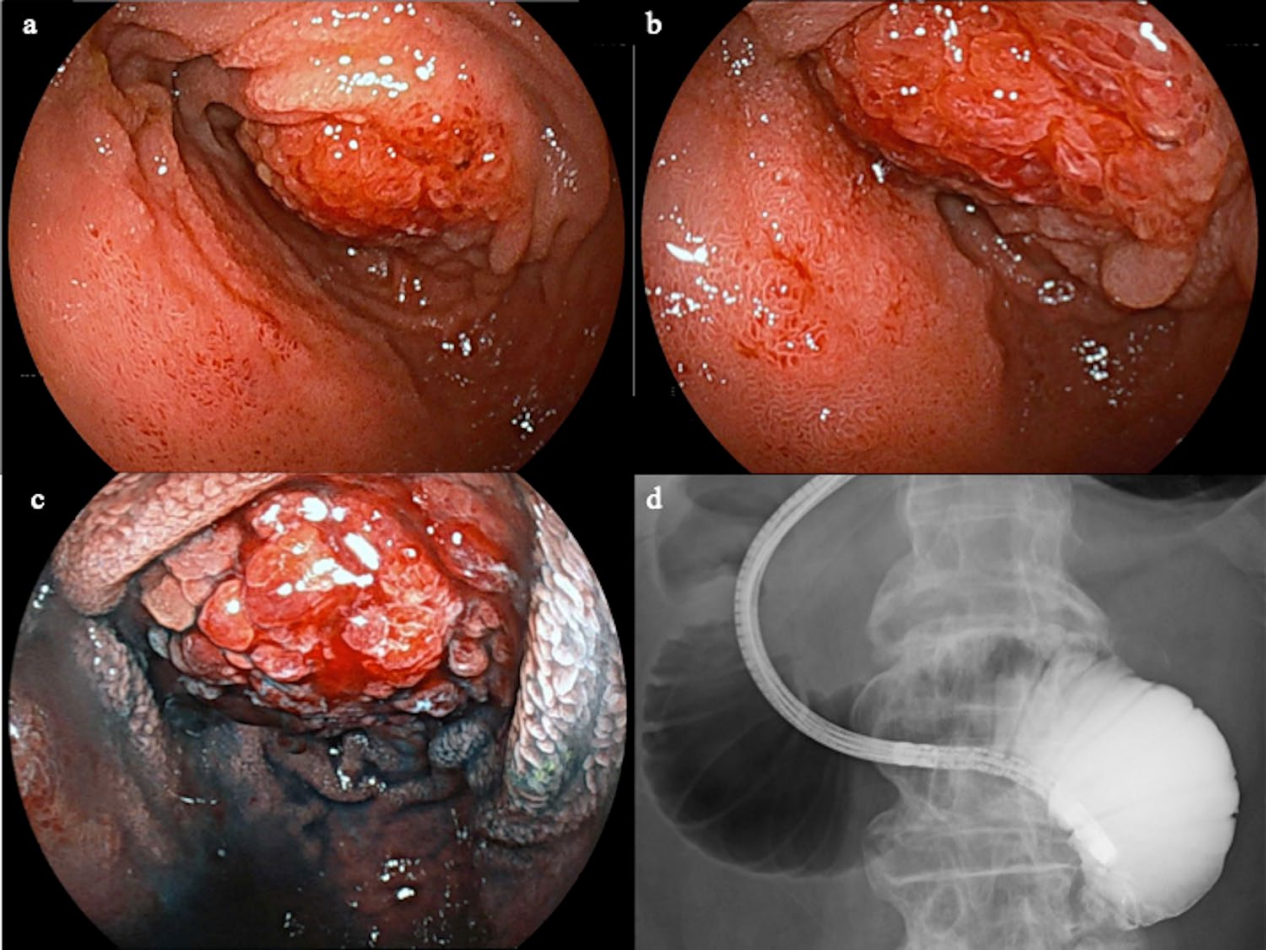
**a**, **b** Double-balloon endoscopy revealed a smooth mass in the jejunum with bleeding and asymmetrical luminal stenosis. **c** Chromoendoscopy with indigo carmine enhanced visualization of enlarged villi. **d **Small intestinal radiography during oral DBE demonstrated jejunal obstruction

Double-balloon endoscopy (DBE) revealed a reddish and friable protrusion with luminal narrowing in the jejunum with luminal stenosis (Fig. 2a, b). Chromoendoscopy with indigo carmine revealed enlarged villous structure in the center of the lesion (Fig. 2c). The scope could not pass the narrowing. Small intestinal radiography during oral DBE also demonstrated jejunal obstruction (Fig. 2d).

Histopathological examination of the biopsy specimens obtained from the villous structure showed non-neoplastic villous epithelium. Because the endoscopic and histologic findings were suggestive of malignant subepithelial tumor, small bowel resection was performed.

**Fig. 3:**
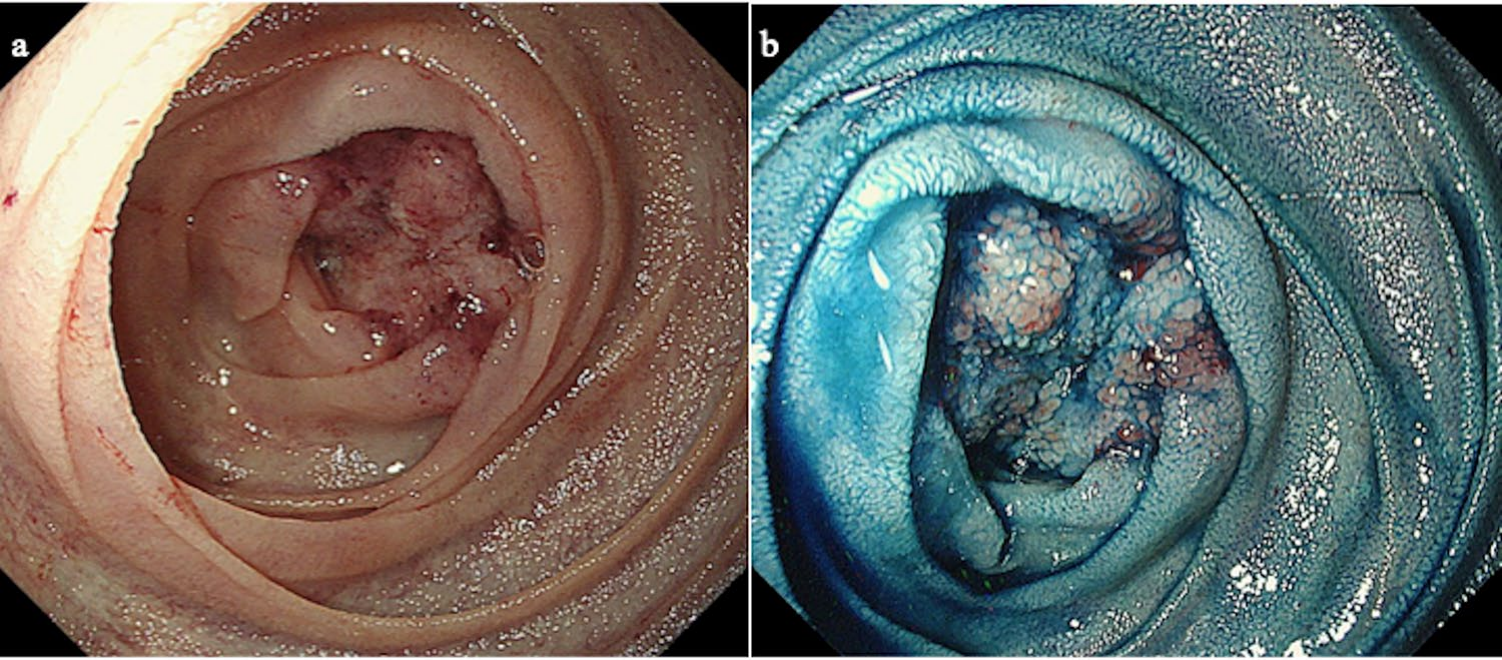
**a **Intraoperative endoscopy from the anal side revealed severe stenosis and fold convergence. **b** Chromoendoscopy using indigo carmine facilitated the irregular margin of the depressed area

**Fig. 4:**
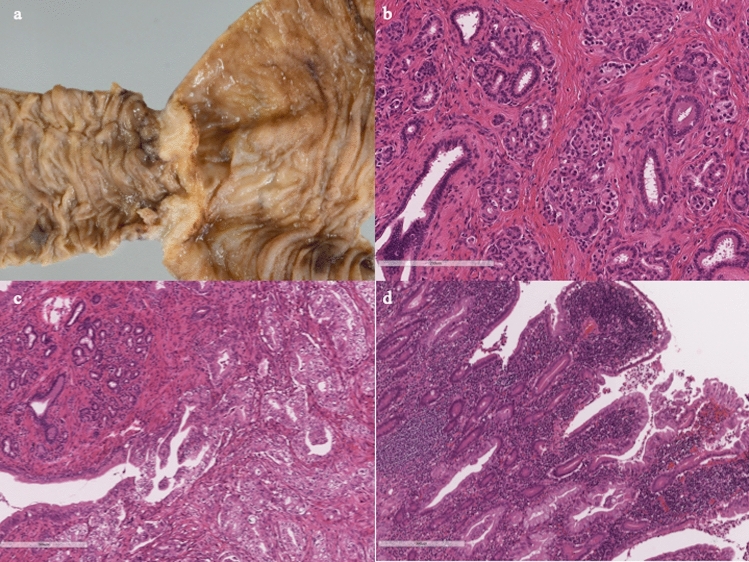
**a** Tumor was a whitish subepithelial lesion-like, measured 15 × 14 mm in size. **b** Histopathological examination of the resected specimen revealed pancreatic tissue in the submucosa, including pancreatic ducts, acinar cells, and islets of Langerhans (Heinrich type I). **c** Histopathological examination revealed moderately differentiated adenocarcinoma, with observed areas of transition from ectopic pancreas to cancer. **d** Inflammatory cell infiltration, mainly consisting of lymphocytes, was observed in the lamina propria

During surgery, it was confirmed that the tumor was located in the jejunum, 30 cm distal to the ligament of Treitz. Intraoperative endoscopy from the anal side of the lesion revealed severe stenosis and fold convergence (Fig. 3a). Chromoendoscopy using indigo-carmine enhanced the irregular margin of the depressed area suggesting malignancy (Fig. 3b). Macroscopically, the tumor was a whitish subepithelial lesion-like, measured 15 × 14 mm in size (Fig. 4a). Histopathological examination of the resected specimen revealed pancreatic tissue in the submucosa, including pancreatic ducts, acinar cells, and islets of Langerhans (Fig. 4b), leading to a diagnosis of ectopic pancreas of Heinrich type I. In addition, it was evident that the tumor contained confluent growth of cancer cells with clear cytoplasm, consistent with moderately differentiated adenocarcinoma (Fig. 4c). The tumor was not regarded as a collision of pancreatic tissue and cancer, because areas of transition from ectopic pancreas to carcinoma were found (Fig. 4c). The center of the tumor was covered by villous hyperplasia with the infiltration of predominant lymphocytes in the lamina propria (Fig. 4d). Based on these findings, the tumor was diagnosed as a cancer arising from ectopic pancreas in the jejunum. Because there was a lymph node metastasized with cancer, the final pathological diagnosis was pT4aN1M0, pStage IIIA according to the TNM classification system for malignant tumors [5].

The patient recovered uneventfully after the surgery, and he has been doing well under an adjuvant chemotherapy with S-1 at a daily dose of 120 mg in accordance with the clinical practice guideline for pancreatic cancer [6].

## Discussion

While ectopic pancreas can occur anywhere within the gastrointestinal tract, the most common site of involvement has been known to be the stomach, followed by the duodenum, the jejunum and the ileum [7, 8]. In most cases, ectopic pancreas is asymptomatic and is incidentally found during endoscopy or autopsy [9]. Even in symptomatic patients, the symptoms are nonspecific, including abdominal pain, bleeding, or vomiting due to obstruction [4]. However, it should be noted that the pancreatic tissue possibly shows malignant transformation, although the incidence ranges from 0.7 to 1.8% [10] among the autopsy cases. Nevertheless, it still remains obscure whether ectopic pancreas has a higher burden for cancer development when compared to the pancreas [11].

In a literature review of 54 cases of cancer originating from ectopic pancreas, Cazacu IM, et al. reported that the majority of the cases were symptomatic, and they occurred in middle-aged men. In addition, there was a trend that the background ectopic pancreas was classified as Heinrich type I and exhibited a subepithelial-like appearance macroscopically [11]. When we attempted an online search of PubMed during a period from 1995 until 2025, we identified only eight cases of pancreatic cancer arising from an ectopic pancreas in the jejunum, including our present case (Table 1) [9, 12–16]. Such a small number of jejunal cancers in the literature may be explained by the rarity of jejunal ectopic pancreas. In those cases, the tumors occurred in middle-aged or elderly patients. While the symptoms were nonspecific and variable, the tumors were associated with increase in serum CA19-9 levels. In addition, four of the eight tumors had lymph node metastasis or peritoneal dissemination, and four tumors occurred from ectopic pancreas of Heinrich type I.

**Table 1:** Published cases of pancreatic cancer arising from an ectopic pancreas in the jejunum

Reference	Age, years, gender	Symptoms	CA 19–9, U/ml	Distance from the ligament of Treitz, cm	Size, mm	Metastasis	Heinrich type
Makhlouf et al. [12]	71, male	None (CT image abnormalities)	Unknown	Unknown	25	None	I
Makhlouf et al. [12]	61, male	Abdominal pain, vomiting	Unknown	8	15	Peritoneum	I
Arao et al. [13]	63, male	Epigastric discomfort and vomiting	6100	Unknown	40	Lymph node and liver	II
Fujita et al. [14]	64, female	Abdominal distension, epigastric pain	Unknown	45	20	Lymph node	Unknown
Song et al. [15]	74, male	Abdominal pain	88.4	Unknown	30	None	I
Yamaoka et al. [9]	69, female	None (CT image abnormalities)	635	Unknown (100 cm from incisor teeth)	28	Lymph node	II
Yamamoto et al. [16]	76, male	Abdominal distention, vomiting	Unknown	20	35	None	II
Our case	74, male	Anorexia, vomiting, abdominal pain	684	30	15	None	I

*CA19-9* carbohydrate antigen 19-9, *CT* computed tomography

It has been reported that ectopic pancreas in the small bowel is observed as a non-specific, SEL-like lesion under endoscopy [17]. In contrast, there have been only limited descriptions regarding the endoscopic features of jejunal cancer arising from ectopic pancreas. In our case, the boundary of the tumor was observed as SEL-like tumor, suggesting a diagnosis of gastrointestinal mesenchymal tumor, malignant melanoma and metastatic small intestinal tumor [14]. Unlike those small bowel tumors, however, the tumor of our patients had central depression with villous hyperplasia and severe jejunal stricture. These findings may be explained by severe fibrosis induced by desmoplastic reaction to cancer cells and, presumably, repeated inflammation occurring in the pancreatic tissue, particularly the lamina propria. In addition to these endoscopic findings, ectopic pancreatic cancer should be considered as a differential diagnosis in a case of small bowel SEL with intestinal obstruction and elevated serum CA19-9.

In conclusion, we could observe cancer arising from ectopic pancreas by way of enteroscopy. Although this condition is extremely rare, SEL-like tumor accompanied by luminal constriction and surface changes may be characteristic of small bowel cancer originating from ectopic pancreas.

## References

[1] Gaspar Fuentes A, Campos Tarrech JM, Fernandez Burgui JL, et al. Pancreatic ectopias. Rev Esp Enferm Apar Dig. 1973;39:255–68. 4699117

[2] Gokhale UA, Nanda A, Pillai R, et al. Heterotopic pancreas in the stomach: a case report and a brief review of the literature. JOP. 2010;11:255–7. 20442522

[3] Hickman DM, Frey CF, Carson JW. Adenocarcinoma arising in gastric heterotopic pancreas. West J Med. 1981;135:57–62. 7257381 PMC1272925

[4] Ulrych J, Fryba V, Skalova H, et al. Premalignant and malignant lesions of the heterotopic pancreas in the esophagus: a case report and review of the literature. J Gastrointestin Liver Dis. 2015;24:235–9. 26114184 10.15403/jgld.2014.1121.242.uly

[5] Brierley JD, Gospodarowicz MK, Wittekind C, editors. The TNM classification of malignant tumours. 8th ed. Wiley Blackwell; 2017.

[6] Okusaka T, Nakamura M, Yoshida M, et al. Clinical practice guidelines for pancreatic cancer 2022 from the Japan pancreas society: a synopsis. Int J Clin Oncol. 2023;28:493–511. 36920680 10.1007/s10147-023-02317-x PMC10066137

[7] Goodarzi M, Rashid A, Maru D. Invasive ductal adenocarcinoma arising from pancreatic heterotopia in rectum: case report and review of literature. Hum Pathol. 2010;41:1809–13. 20869744 10.1016/j.humpath.2010.06.005

[8] Stock C, Keutgen XM, Pisapia D, et al. Heterotopic pancreatic neoplasm presenting as an obstructing mass at the fourth portion of the duodenum. JOP J Pancreas. 2011;12:241–3. 21546699

[9] Yamaoka Y, Yamaguchi T, Kinugasa Y, et al. Adenocarcinoma arising from jejunal ectopic pancreas mimicking peritoneal metastasis from colon cancer: a case report and literature review. Surg Case Rep. 2015;1:114. 10.1186/s40792-015-0118-1. 26943438 10.1186/s40792-015-0118-1 PMC4648850

[10] Guillou L, Nordback P, Gerber C, et al. Ductal adenocarcinoma arising in a heterotopic pancreas situated in a hiatal hernia. Arch Pathol Lab Med. 1994;118:568–71. 8192567

[11] Cazacu IM, Luzuriaga Chavez AA, et al. Malignant transformation of ectopic pancreas. Dig Dis Sci. 2019;64:655–68. 30415408 10.1007/s10620-018-5366-z

[12] Makhlouf HR, Almeida JL, Sobin LH. Carcinoma in jejunal pancreatic heterotopia. Arch Pathol Lab Med. 1999;123:707–11. 10420228 10.5858/1999-123-0707-CIJPH

[13] Arao J, Fukui H, Hirayama D, et al. A case of aberrant pancreatic cancer in the jejunum. Hepatogastroenterology. 1999;46:504–7. 10228851

[14] Fujita K, Hirakawa K, Matsumoto T, et al. Small-intestinal cancer arising from heterotopic pancreas. Endoscopy. 2008;40:E240–1. 18991219 10.1055/s-2008-1077693

[15] Song JY, Han JY, Choi SK, et al. Adenocarcinoma with intraductal papillary mucinous neoplasm arising in jejunal heterotopic pancreas. Korean J Pathol. 2012;46:96–100. 23109987 10.4132/KoreanJPathol.2012.46.1.96 PMC3479704

[16] Yamamoto K, Ishimori T, Okada T, et al. Small bowel intussusception due to adenocarcinoma of ectopic pancreas in the jejunum: a case report. Surg Case Rep. 2024;10:264. 10.1186/s40792-024-02060-z. 39557711 10.1186/s40792-024-02060-z PMC11573949

[17] Kida M, Kawaguchi Y, Miyata E, et al. Endoscopic ultrasonography diagnosis of subepithelial lesions. Dig Endosc. 2017;29:431–43. 28258621 10.1111/den.12854

